# Synthetic ablations in the *C. elegans* nervous system

**DOI:** 10.1162/netn_a_00115

**Published:** 2020-03-01

**Authors:** Emma K. Towlson, Albert-László Barabási

**Affiliations:** Network Science Institute and Department of Physics, Northeastern University, Boston, MA, USA; Media Laboratory, Massachusetts Institute of Technology, Cambridge, MA, USA; Network Science Institute and Department of Physics, Northeastern University, Boston, MA, USA; Department of Medicine, Brigham and Women’s Hospital, Harvard Medical School, Boston, MA, USA; Department of Network and Data Science, Central European University, Budapest, Hungary

**Keywords:** *C. elegans*, Graph theory, Network control theory, Neuronal ablations, Synthetic lethality

## Abstract

Synthetic lethality, the finding that the simultaneous knockout of two or more individually nonessential genes leads to cell or organism death, has offered a systematic framework to explore cellular function, and also offered therapeutic applications. Yet the concept lacks its parallel in neuroscience—a systematic knowledge base on the role of double or higher order ablations in the functioning of a neural system. Here, we use the framework of network control to systematically predict the effects of ablating neuron pairs and triplets on the gentle touch response. We find that surprisingly small sets of 58 pairs and 46 triplets can reduce muscle controllability in this context, and that these sets are localized in the nervous system in distinct groups. Further, they lead to highly specific experimentally testable predictions about mechanisms of loss of control, and which muscle cells are expected to experience this loss.

## Author Summary

“Synthetic lethality” in cell biology is an extreme example of the effects of higher order genetic interactions: The simultaneous knockout of two or more individually nonessential genes leads to cell death. We define a neural analog to this concept in relation to the locomotor response to gentle touch in *C. elegans*. Two or more neurons are synthetic essential if individually they are not required for this behavior, yet their combination is. We employ a network control approach to systematically assess all pairs and triplets of neurons by their effect on body wall muscle controllability, and find that only surprisingly small sets of neurons are synthetic essential. They are highly localized in the nervous system and predicted to affect control over specific sets of muscles.

## INTRODUCTION

“Synthetic lethality” (Nijman, 2011), a term well established in cell biology, refers to the phenomenon whereby the deletion of individual genes is tolerated by an organism, but the deletion of the combination is lethal. Such synthetic lethal pairs are of particular interest because of their importance for cancer therapies (Kaelin, 2005; Nijman, 2011; O’Neil, Bailey, & Hieter, 2017). Double or higher order gene deletions can also affect the growth rate or other quantitative traits of a cell (Costanzo et al., 2019), and they are of particular interest if the outcome is not explained by the simple summation of the phenotypes of the individual knockouts (Tarailo, Tarailo, & Rose, 2007). Studies in yeast (Saccharomyces cerevisiae; Scherens & Goffeau, 2004) have found that digenic interactions tend to be enriched with single genes known to affect fitness; that is, most are positive or negative interactions of essential genes (Costanzo et al., 2016; Tong et al., 2001). Trigenic interactions have been shown to be weaker in magnitude than digenic interactions, and spread across many different genes/distant bioprocesses, although functionally related genes are hubs on the trigenic network (Haber et al., 2013; Kuzmin et al., 2018).

Taken together, double and higher knockouts have offered a systematic tool to explore cellular systems, and have led to multiple mechanistic insights, as well as therapeutic applications. Despite its widespread use and value in cell biology, the systematic study of higher order neural ablations is lacking in neuroscience. Certainly, many studies in *C. elegans* have investigated the effects of single neuronal *class* ablations (two to 13 individual neurons), and much fewer the effects of two or more class ablations (Fang-Yen, Gabel, Samuel, Bargmann, & Avery, 2012). These simultaneous ablations tend to be small sets of neurons targeted by a precise hypothesis or query, and in the context of specific functions of interest. Early work includes probing the circuitry behind touch sensitivity (Chalfie et al., 1985; Chalfie & Sulston, 1981), finding, for example, that the simultaneous ablation of AVA and AVD leads to a loss of the worm’s ability to move backwards, an effect not observed with the ablation of either class alone. Such experiments were collectively able to identify two pathways for anterior touch–induced locomotion, and one for posterior touch. Multiple ablations within the pharynx revealed the surprising result that no neuron in the pharyngeal nervous system is necessary for pharyngeal pumping, and only one (M4) is required for the animals to grow into fertile adults (Avery & Horvitz, 1989). Further, the pharyngeal neurons may be divided into three functional groups based on the behavioral effects of ablating them. A study that systematically ablated combinations of chemosensory neurons demonstrated that the simultaneous ablation of the four classes ADF, ASG, ASI, and ASJ produces larval worms that enter the dauer stage—a specialized form resistant to harsh conditions—regardless of environment (Bargmann & Horvitz, 1991). Smaller subsets of these neuronal ablations did not lead to the same effect, revealing the functional interdependence of these classes. Ablation of the AVL and DVB neurons, individually and as a pair, is moderately to severely detrimental to the defecation cycle, with the most pronounced effect produced by the double ablation (McIntire, Jorgensen, Kaplan, & Horvitz, 1993). This highlights an amount of redundancy in their function, and given the dependence of enteric muscle contractions on these GABA-ergic neurons, provides evidence that GABA may be a *stimulatory* as well as inhibitory neurotransmitter.

Much of the reason for such carefully targeted and class-based queries—instead of systematically exploring the whole space of individual neurons—is the tractability of performing so many experiments. Computational and theoretical approaches do not have this limitation and have therefore allowed for more extensive lesion-based analyses. A number of studies into mammalian brain networks have focused on lesioning hubs, showing that in the cat and macaque brains these nodes have the biggest impact on network structure (Sporns, Honey, & Kötter, 2007) and provide structural robustness (Kaiser, Martin, Andras, & Young, 2007). These macroscale studies have implications for understanding the integrated and segregated organization and dynamics of the human brain (Alstott, Breakspear, Hagmann, Cammoun, & Sporns, 2009; de Reus & van den Heuvel, 2014), and injuries or diseases such as traumatic brain injury, stroke, and Alzheimer’s (Aerts, Fias, Caeyenberghs, & Marinazzo, 2016). In *C. elegans*, in silico ablation has been used to validate computational models and to make new predictions about neuronal roles (Roberts et al., 2016). With its tractable size and well-characterized wiring diagram, *C. elegans* provides a unique opportunity for whole-brain simulation (Kim, Leahy, & Shlizerman, 2019; Kim, Santos, Alkema, & Shlizerman, 2019). Simulations are becoming increasingly important in neuroscience, including for the study of neurosensory integration. Within such simulations, AVB ablation has been shown to lead to reduced neuronal activity patterns (Kim, Leahy, et al., 2019) and altered response modes (Kunert, Shlizerman, & Kutz, 2014), consistent with the experimental finding that its removal impedes forward locomotion (Chalfie et al., 1985). Neuroanatomical models are able to replicate the experimental observation (Ino & Yoshida, 2009) that ablating the chemosensory neurons AFEL/R results in a deterioration in klinotaxis performance (Izquierdo & Beer, 2013; Izquierdo & Lockery, 2010). Integrating experimental and computational ablations has also led to new insights into the structure of the nervous system. For example, one study ablated combinations of the command interneurons and ASH (sensory neurons associated with harsh touch) and compared results with in silico ablations of the same sets of neurons (Rakowski, Srinivasan, Sternberg, & Karbowski, 2013). This approach was able to predict the likely polarities of connections by matching the computational model to experimental observations. Purely network-based studies have found a vulnerability to hub lesioning similar to other brain networks (Iyer, Killingback, Sundaram, & Wang, 2013). One network study uses a concept of network flow to partition the nervous system into communities, and assess the effect of single and double neuronal ablation on these communities (Bacik, Schaub, Beguerisse-Díaz, Billeh, & Barahona, 2016). This approach is able to recover neurons with integrative roles such as AIA and predict pairs that disrupt the robustness of partitions.

It is likely that systematic data on the outcome of double and higher ablations could offer a resource as useful to the community as higher order knockouts are to cell biology. In this paper we set out to do just that—use the tools of network control to offer a complete set of predictions on double and triple ablations’ effect on locomotion. Indeed, a comprehensive set of theoretical predictions could also guide future experiments and shed light on mechanistic network effects in neural circuits. The predictions can expose network-level redundancy and robustness, and potentially even guide restoration of lost function. The emergence of genetic ablation methodologies and precise optical targeting (Leifer, Fang-Yen, Gershow, Alkema, & Samuel, 2011) promises tools that may make systematic double and triple ablations possible in the near future.

**Figure 1.**
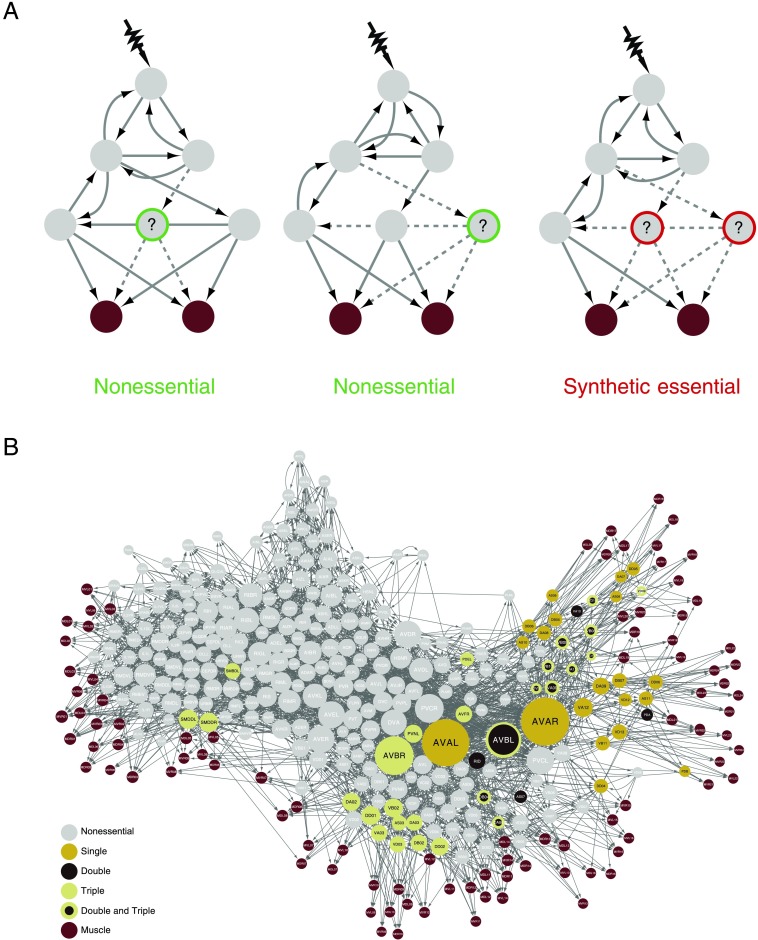
Synthetic essentiality in double and triple neuronal ablations. (A) Individual neurons are systemically ablated from the wiring diagram, and the impact on muscle controllability assessed. Neurons that do not lead to a loss of control are deemed “nonessential” (left and middle). In a double ablation, two neurons may be nonessential individually, but when they are both ablated, we predict a reduction in the number of controllable muscles and the pair is essential. We define this as a synthetic essential pair (right). (B) Predicted essential single, pairs of, and triplets of neurons in the *C. elegans* connectome. Mustard nodes denote individual neurons predicted to be essential as per Yan et al. (2017). Neurons involved in a pair predicted to be synthetic essential are colored dark brown, and those in a triplet are colored yellow-green; if a neuron is part of a pair and a triple, it is colored dark brown and yellow-green. Muscles are in red-brown and nonessential neurons in gray, and all cells are sized by nodal degree.

Synthetic lethality is necessarily defined in relation to some phenotype: whether a genetic component is essential to the function in question. In single-cell organisms, the metric of fitness is usually growth or lethality. In neural systems, one could consider multiple such phenotypes. Here, we illustrate the value of systematic ablation studies in neuroscience by exploring the space of neuronal functional interactions in the *C. elegans* nervous system and their role in locomotion. This is made possible by the development of network control (Betzel, Gu, Medaglia, Pasqualetti, & Bassett, 2016; Gao, Liu, D’Souza, & Barabási, 2014; Liu & Barabási, 2016; Tang & Bassett, 2017; Yan, Ren, Lai, Lai, & Li, 2012), a theoretical framework that helped frame the locomotor response of *C. elegans* to sensory input as a target control problem (Chew et al., 2017; Towlson et al., 2018; Yan et al., 2017). This helped to identify 20 neurons that when ablated individually are predicted to lead to a loss of muscle control in the behavioral response to gentle touch; see Supplementary Table S3. We hypothesized that because of network effects, there would be nonessential neurons as categorized by this approach that, when simultaneously ablated, would have an impact on controllability of the muscles. This *pair* (or *triplet*) would then be essential for locomotion; see Figure 1A. Therefore, using the same theoretical framework, here we systematically ablated in silico each possible neuronal pair, and each possible neuronal triplet, and examined the effects, if any, on muscle controllability following external input to mechanosensory neurons. Our results point to highly localized, and highly specific, double and triple neuronal interactions in locomotor control (Figure 1B). We identify the small groups of muscles affected by these interactions, together with the few network-level mechanisms behind the loss of control.

## RESULTS

For each possible pair and triplet of neurons, we performed a structural controllability analysis with these neurons and their connections removed from the wiring diagram. We employed a target control approach, with the mechanosensory neurons for gentle touch as input nodes ({ALML, ALMR, AVM} for anterior touch, or {PLML, PLMR} for posterior touch) and the 95 body wall muscle cells as output nodes (see Methods). This allowed us to quantify the number of muscles that can be independently controlled via the application of suitable input signals to the input neurons. Any deviation from the healthy case signals an impact on controllability caused by the ablation. Given that there are 279 nonpharyngeal neurons in the *C. elegans* nervous system (not excluding any sensory input for the purposes of this illustrative calculation), we have a set of 279 neurons from which to draw neurons for candidate ablations. We therefore tested ^279^*C*_2_ = 38,781 double neuronal ablations, and ^279^*C*_3_ = 3,580,779 triple neuronal ablations.

### Synthetic Pairs

We find that 5,428 of the 38,781 possible double ablations lead to a loss of control over the muscles in the locomotor response to gentle touch, while the remaining 33,353 have no effect on locomotion; see Table 1 and Supplementary Tables S1 and S4. Of the 5,428 pairs that do affect controllability, 190 comprise neuron pairs whose members were already predicted (and validated) to be essential via single ablations (E_single_E_single_), 5,180 contain one previous neuron and one individually nonessential (E_single_N_single_), and one is the trivial case of the removal of the pair of input neurons in the case of posterior touch (meaning no external control signal is received by the network). The remaining 57 pairs have no overlap with the set of predictions from single ablations (N_single_N_single_), and thus constitute synthetic essential pairs. They involve 16 distinct neurons, most of which are ventral motor neurons, and are predicted to affect a small set of muscles in the ventral and/or dorsal posterior section of the body (see Supporting Information, Section III).

**Table 1.** Double ablation predictions and relation to single ablation predictions. A selected pair of neurons may comprise two individually essential neurons (E_single_), one essential and one nonessential neuron (N_single_), or two nonessential neurons. Synthetic essential pairs with no overlap with single ablation predictions are colored red. Totals exclude the trivial case of removal of all input neurons.

	Reduction	No effect
E_single_E_single_	190	0
E_single_N_single_	5,180	0
N_single_N_single_	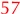	33,353

We also note a single case of enhancement of control loss, that is, a negative interaction (see Supporting Information, Section I). In genetic knockouts, a *negative* interaction enhances the detrimental effect of a knockout, while a *positive* interaction diminishes it. AS11 is predicted to be essential individually, leading to a reduction in control from 89 to 88 muscles. (Note that the AS class as a whole has been experimentally implicated in locomotory coordination; Tolstenkov et al., 2018.) When RID is simultaneously ablated, we predict that this number further reduces to 87 independently controllable muscles.

Interestingly, for each ablated neuron, the number of independently controllable muscles reduces by a maximum of one. Given that 89 muscles are found to be controllable in the healthy worm (Yan et al., 2017), a double ablation leads to at most a reduction of two, to 87 controllable muscles.

### Mechanisms

Neuronal ablations affect controllability by changing the number of linearly independent control signals that arrive at the muscles from the input neurons. In other words, the graph theoretic criterion for a neuron (or set of neurons) to be essential is that it alters the number of pathways of unique length between the input and output nodes (Gao et al., 2014). In our original study, we demonstrated that single ablations can achieve the same effect by reducing the numbers of the sets of motor neurons directly connecting to sets of muscles (Yan et al., 2017). Here, we find that double ablations act one layer higher—they reduce the number of linearly independent control signals received by the motor neurons, causing a knock-on effect of further constricting the possible number that can ultimately arrive at the muscles.

**Figure 2.**
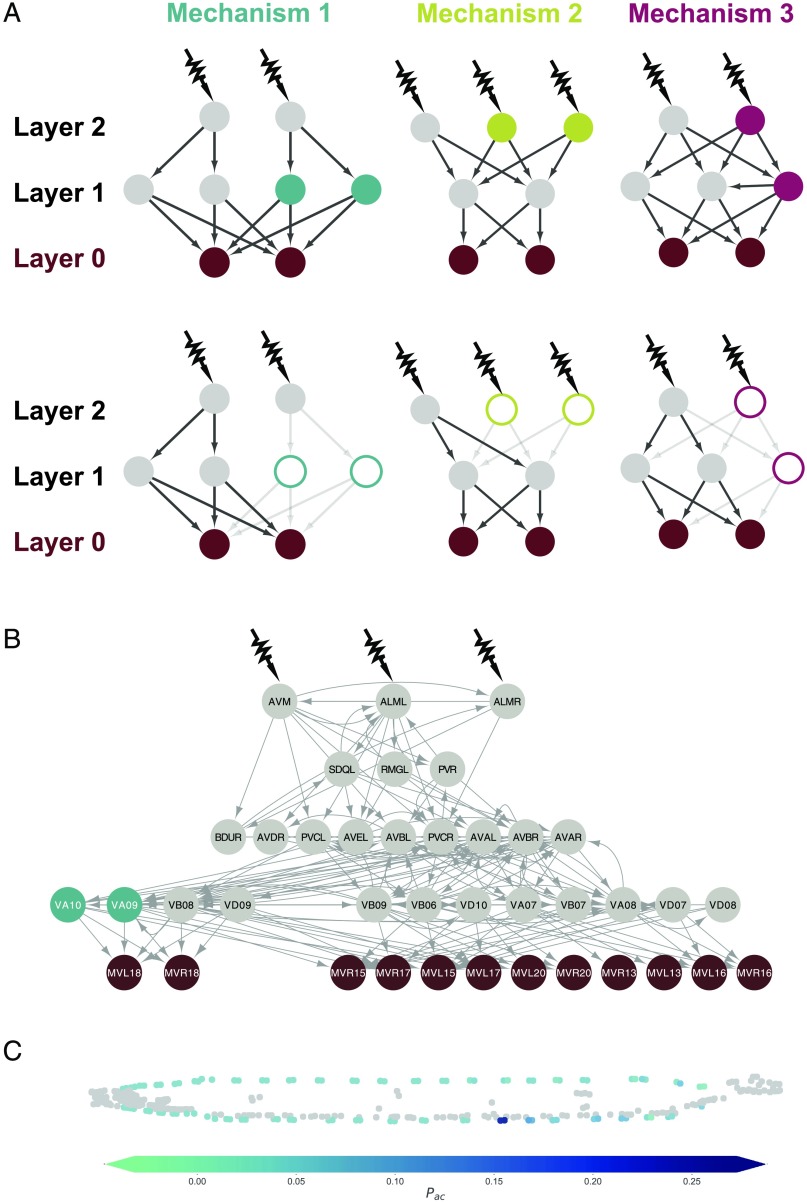
Control mechanisms behind the synthetic essentiality of neuronal pairs. (A) Three distinct mechanisms are observed for the reduction in control found in synthetic essential pairs. Forty pairs are predicted to lose fine muscle control via Mechanism 1, one by Mechanism 2, and 16 by Mechanism 3. Mechanism 1: (Upper) Since there are more Layer 1 neurons receiving independent control signals than there are muscles, the muscles are controllable. (Lower) The removal of two Layer 1 neurons disconnects the pathway from one of the Layer 2 neurons to the muscles. Mechanism 2: (Upper) Since there are the same number of Layer 1 neurons with independent control signals as there are muscles, the muscles are controllable. (Lower) The removal of two Layer 2 neurons leaves only one independent control signal arriving at Layer 1, as in Mechanism 1. Only one muscle may be independently controlled. Mechanism 3: (Upper) Since there are more Layer 1 neurons receiving independent control signals than there are muscles, the muscles are controllable. (Lower) The removal of one Layer 1 and one Layer 2 neuron causes there to be only one independent control signal arriving at the two neurons in Layer 1, and consequently only one independent control signal arriving at the muscles. Again, only one independent control arrives at Layer 1, and thus also the muscles. (B) Example synthetic essential pair, VA09 and VA10. This double ablation is predicted to affect control of the two muscles MVR18 and MVL18 via Mechanism 1. (C) The probability of ablation-induced loss of control over each muscle in the body of *C. elegans* following ablation of the pair {VA09, VA10}. Muscles most likely to lose control are colored dark blue, and the least likely to lose control are colored green. The locations of neuron cell bodies are shown in gray.

**Figure 3.**
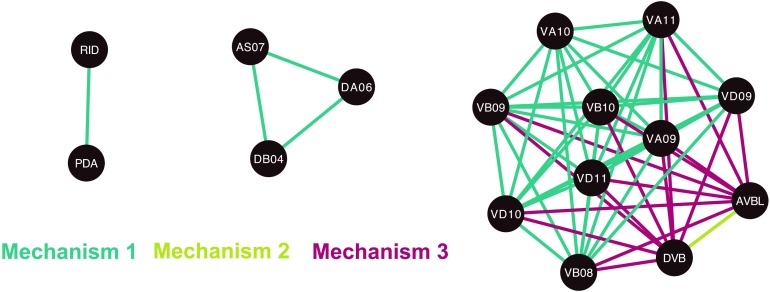
Neurons involved in synthetic essentiality via double ablations. The neurons comprising pairs of synthetic essential neurons are shown as networks, in which two neurons are linked if they occur together in a synthetic essential pair. Edge color describes the mechanism of essentiality.

To be specific, we identified only three distinct mechanisms that lead to a loss of control over body wall muscles in the response to gentle touch for all of the double ablations (see Figure 2A and Figure 3). Let us label the muscles as Layer 0 cells, and we label any neuron with a direct connection to a muscle a Layer 1 neuron. Finally, neurons that are a path length of two away from a muscle form Layer 2, and so on, such that the layer number of a neuron is the length of the shortest path from that neuron to a muscle cell. Figure 2A illustrates the nature of the three mechanisms that lead to loss of control in synthetic essential neuron pairs.

#### Mechanism 1 (40 synthetic essential pairs).

Figure 2A first shows a network with two Layer 0 nodes, four Layer 1 nodes, and two Layer 2 nodes. In this network, the Layer 2 nodes do not fully connect to the Layer 1 nodes, rather splitting them into two groups of two, each of which receives one control signal. The removal of two nodes from one of these groups disconnects one control signal, and control is lost over one of the Layer 0 nodes.

Figure 2B illustrates the ablation of the pair {VA09, VA10}, which we find to display synthetic essentiality via this mechanism. The two muscle cells MVL18 and MVR18 connect to only four neurons, VA09, VA10, VB08, and VD09. These four neurons are likely to receive only three independent control signals, meaning correlation is expected in their activity levels. If either VA09 or VA10 is ablated, at least two independent control signals remain, which is enough to control the two muscles. But if both VA09 and VA10 are ablated, it is likely that only one independent control signal remains, and control is lost over one of the muscle cells MVL18 or MVR18.

#### Mechanism 2 (1 synthetic essential pair).

The second network in Figure 2A has two Layer 0 cells, two Layer 1, and three Layer 2 nodes. If we remove a Layer 2 neuron, there are still enough independent control signals received by the Layer 1 nodes to control the muscles in Layer 0. But if we remove a second Layer 2 neuron, only one control signal is received by the two Layer 1 neurons, and consequently only one Layer 0 neuron can be controlled. The pair that is governed by this mechanism is {AVBL, DVB}.

#### Mechanism 3 (16 synthetic essential pairs).

The third network in Figure 2A has two muscle cells, which connect to three Layer 1 neurons, which receive input from two Layer 2 neurons. Since there are more Layer 1 neurons—each of which receives a linearly independent signal—than muscles, each of the muscles is independently controllable (Yan et al., 2017). If one Layer 1 neuron is removed, the muscles are still controllable, as there is still one independent signal from each Layer 1 neuron for each one muscle. But if a Layer 2 neuron is *also* removed, only one independent control signal arrives at the two Layer 1 neurons. This means there is only one independent control signal to control two muscles, and control is therefore lost over one of them. Synthetic essential pairs governed by Mechanism 3 comprise either AVBL or DVB and a ventral motor neuron, such as {AVBL, VB10} and {DVB, VD10}.

We identified only 57 synthetic essential pairs, which we predict to lead to loss of control over the body wall muscles in the behavioral response to gentle touch via one of three distinct mechanisms. These mechanisms are rooted one layer up from the muscles, acting to reduce the number of independent control signals received by the motor neurons (and then consequently the muscles). We note that more mechanisms could exist in an arbitrary network, but only these three are observed in the *C. elegans* nervous system. Many of the neurons involved are ventral cord motor neurons, and the synthetic essential pairs predict their functional interdependence.

### Synthetic Triplets

Of all the 3,580,779 possible triple ablations, 2,847,827 (79.5%) are predicted to have no effect and 732,952 are predicted to lead to a loss of muscle control in the behavioral response to gentle touch. Of these essential triplets, 731,525 are explained through the simple summation of effects of essential individual neurons and synthetic essential pairs; see Table 2 and Supplementary Tables S2 and S5. Only the remaining 46 triplets, and 1,381 negative interactions, exhibit synthetic essentiality, a remarkably small number. These synthetic essential triplets comprise 28 distinct neurons and are grouped into four groups based on the set of muscle cells they impact (which comprise one, two, four, or eight muscles). Again, we find that the number of independently controllable muscles reduces by a maximum of one for each ablated neuron, and hence we observe a reduction of no more than three (leaving 86 controllable muscles) following a triple ablation. We observe negative interactions, that is, a greater loss of control than expected by the simple summation of the loss due to the single and double ablations, in 1,381 cases (see Supporting Information, Section II); 1,363 triplets contain only one essential neuron, yet lead to a reduction in control of two muscle cells, and 18 contain two essential neurons and lead to a reduction in control of three muscle cells.

**Table 2.** Triple ablation predictions and relation to double and single ablation predictions. A selected triplet of neurons is composed of combinations of individually essential neurons (E_single_), individually nonessential neurons (N_single_), and synthetic essential pairs of neurons (E_pair_). Synthetic essential triplets with no overlap with single ablation predictions, and not containing a complete pair of synthetic essential neurons, are colored red. Totals exclude the trivial case of removal of all input neurons.

		Reduction	No effect
Pairs	3E_pair_	149	0
	2E_pair_	16	0
	E_pair_E_single_	44	0
	E_pair_N_single_	14,170	0
No pairs	E_single_E_single_E_single_	1,140	0
	E_single_E_single_N_single_	49,210	0
	E_single_N_single_N_single_	668,176	0
	N_single_N_single_N_single_	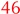	2,847,827

### Mechanisms

**Figure 4.**
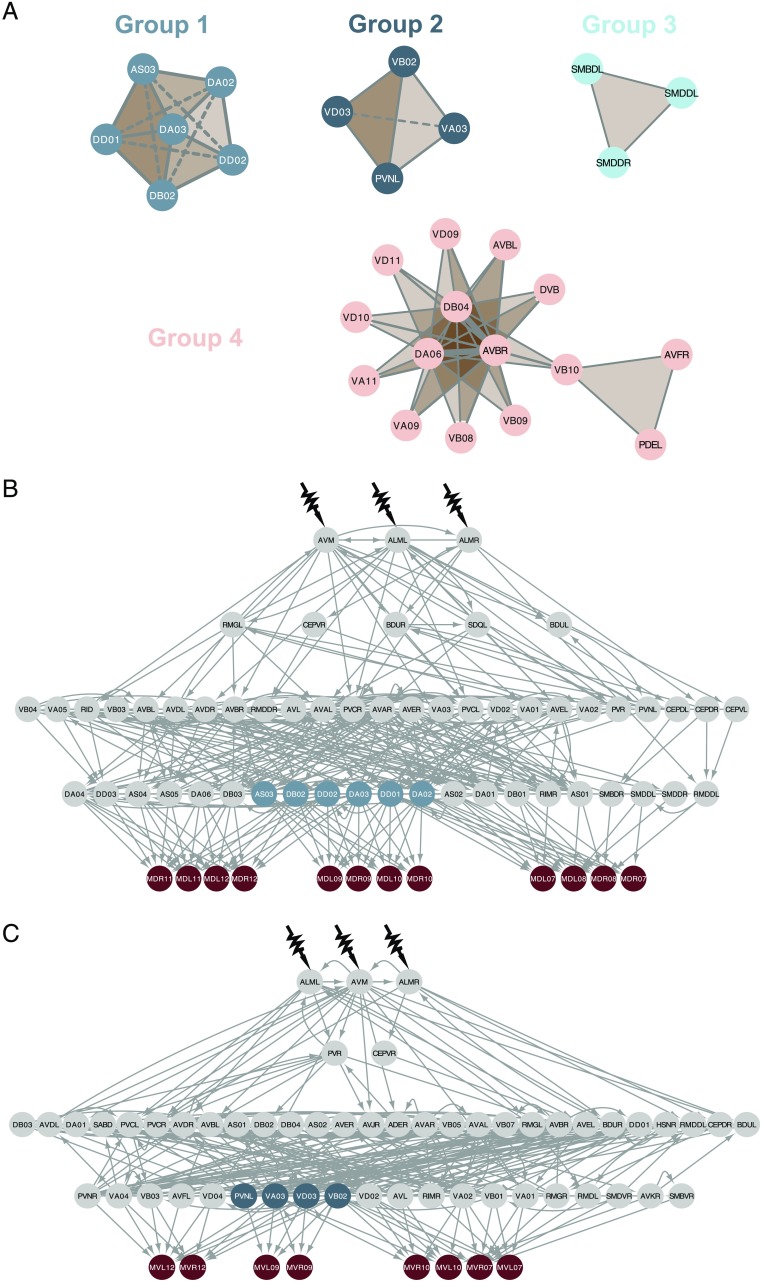
Groups of neurons involved in synthetic essentiality via triple ablations. (A) The neurons comprising triplets of synthetic essential neurons can be divided into four groups, of sizes six, four, three, and 15 neurons respectively. In the depicted networks, two neurons are linked if they occur together in a synthetic essential triplet, and edge width increases with the number of such triplets. Each entire triplet of three is a simplicial complex, shown as a shaded triangular face. In Groups 1–3, any selection of three neurons from the group constitute a predicted synthetic essential triplet. This is not the case for Group 4, which comprises 21 triplets that have at least one neuron in common with each other. (B) Group 1 and the relevant control pathways. The set of muscle cells {MDL09, MDR09, MDL10, MDR10} connect to the nervous system via neuromuscular junctions to only the six neurons in Group 1. To control each of these muscles independently requires four independent control signals, one for each muscle cell. Thus, the removal of any three of the neurons in Group 1 leads to at most three independent control signals arriving at the muscles, and a maximum of three independently controllable muscles. (C) Group 2 and the relevant control pathways. Similar to the mechanism in (B), the ablation of any three of the neurons {PVNL, VA03, VD03, VB02} leads to a loss of independent control over the muscle set {MVL09, MVR09}.

We identified four distinct groups of neurons within the synthetic essential triplet predictions; see Figure 4 and Supplementary Figure S1. Within Groups 1 and 2, one of six neurons and one of four, any combination of three neurons from the set leads to a predicted loss of control. The abstracted group networks of triplets are simplicial complexes (Giusti, Ghrist, & Bassett, 2016; Sizemore, Phillips-Cremins, Ghrist, & Bassett, 2019; Figure 4A). In networks, nodes are connected pairwise via links that represent dyadic relationships. Yet, polyadic relationships, in which three or more nodes interact simultaneously, abound in real systems. In algebraic topology, these generalized connections are called simplexes, and the system as a whole, a simplicial complex. Triplets can be described as 2-simplexes, and the essential pairs in Figure 3 as 1-simplexes. To understand the mechanistic origins of these triplets, we turn back to the network. In each case, a set of *N* neurons directly connects to *N* − 2 muscles—which have no other connections to other neurons. Thus, by ablating any choice of three neurons from the set, a maximum number of *N* − 3 independent control signals can reach the *N* − 2 muscles from the sensory neurons, and we lose controllability of one of them. For example, the four muscle cells {MDL09, MDR09, MDL10, MDR10} are connected to the nervous system via six neurons; see Figure 4B. If we remove, say, AS03, DA02, and DA03, only three neurons provide the path to the muscles. This means that only three control signals can reach four muscles, and they can no longer each be independently controlled. Group 3, comprising three neurons, leads to a trivial disconnection of a muscle cell. Group 4 represents an overlap with synthetic essential pairs; see below.

#### Group 1 (20 synthetic essential triplets).

Any selection of three neurons from the set of six {AS03, DA02, DA03, DB02, DD01, DD02} will reduce the control over a group of four muscle cells, {MDL09, MDR09, MDL10, MDR10}, in the response to gentle touch. Given that these six neurons provide the only connections between these four muscles and the nervous system, a maximum of six independent control signals can reach them. When three are ablated, this number is reduced to three independent control signals, one less than the number of muscle cells.

#### Group 2 (4 synthetic essential triplets).

Any selection of three neurons from the group of four {PVNL, VA03, VB02, VD03} leads to a predicted reduction in fine control over two muscle cells, {MVL09, MVR09}, in the response to gentle touch; see Figure 4C.

#### Group 3 (1 synthetic essential triplet).

When the group of three neurons {SMBDL, SMDDL, SMDDR} are ablated, the muscle MDR06 is disconnected entirely from the network. See Supporting Information, Section II.

#### Group 4 (21 synthetic essential triplets).

The 15 unique neurons in this group overlap with the synthetic essential pairs in terms of individual components, but never including a complete pair. They also impact the same muscle cells, with eight of the ten posterior cells affected by the synthetic essential pairs predicted to be affected. Unlike the first three groups, not any combination of three will suffice, yet clear patterns emerge: 20 of the 21 triplets contain AVBR, and either DA06 or DB04, and are predicted to impact a small set of dorsal muscles in the midsection of the worm. These triplets are then completed by one of 10 individual neurons (see Supplementary Table S5)—any of the 10 with each of the pairs {AVBR, DA06} or {AVBR, DB04}. The final prediction {VB10, PDEL, AVFR} links to the rest of the group only via one of the 10 individual neurons (VB10).

Groups 1–3, which comprise only neurons not involved at all in single or double ablations, uncover small groups of muscles with only a few connections to the nervous system (indeed, only via these precise neurons), which are therefore susceptible to loss of control from mechanosensory input following damage to these groups. Given the repeating connectivity patterns observed in the motor system, this is perhaps not surprising (Haspel & O’Donovan, 2011, 2012), and increasing numbers of ablations might expect to uncover further units. Group 4 contains neurons that are also involved in synthetic essential pairs, and centers on the command interneuron class AVB and a number of posterior dorsal and ventral motor neurons. Their appearance together suggests a more complex functional interdependence.

## DISCUSSION

The most striking aspect of the synthetic essential neuronal pairs and triplets predicted above is their level of specificity. Our methodology identifies highly localized groups of neurons and affected muscles, with consistent network-level mechanisms of loss of control following external mechanosensory input. The small size of these subsets, comparable to that of single ablations, is in contrast to studies in the genetic realm that, unsurprisingly given the combinatorics, find many orders of magnitude more essential digenic and trigenic interactions than singly essential genes. Indeed, the trigenic network tends to be ∼100 times larger than the digenic network—itself containing ∼1,000 times more essential combinations than there are singly essential genes. Further, we observe only very few cases of negative interactions and no positive interactions, the dominant results in genetic interactions (Costanzo et al., 2016; Tong et al., 2001). About 83% of budding yeast genes are nonessential, whereas the control framework predicts that about 93% of neurons are nonessential. Double and triple knockout studies have found that 2.3% of double and 1.6% of triple genetic interactions are negative. We predict that only 0.0003% of double neuronal ablations lead to a negative interaction, and 0.04% of triple ablations. The effects of genetic interactions are found to be weaker in magnitude with the addition of more knockouts, yet the phenotypes predicted following synthetic essential neuronal ablations will be similarly different to the healthy worm, given the loss of control over one muscle cell. However, it is not clear whether these differences are significant or meaningful. The analysis we present here is based on structural control, which provides discrete results rather than the continuous measurements accompanying genetic knockouts. This translates to a binary decision—control is lost or not—and limited measurements of relative strength. Structural control also does not allow for the possibility of positive interactions: A second or third ablation will not increase muscle controllability.

Yet, understanding and cataloging the effects of multiple ablations can inform *rescue*. In genetics, the “Lazarus effect” is the counterintuitive restoration of lost function through gene deletion (Motter, Gulbahce, Almaas, & Barabási, 2008). In neural systems, this translates to the possibility of restoring function via neuronal ablation. If neuronal damage has led to the loss of control of a function, such as locomotion, we can seek to restore the function by removing one or more further neurons. According to structural control, we cannot recover control through the removal of neurons and/or links. However, if the extension is made to a more sophisticated analysis incorporating link weights and energetic considerations (Betzel et al., 2016; Yan et al., 2012), we might be able to find such synthetic rescue combinations. Indeed, it is theoretically possible to reduce the energy required to reach certain configurations of the behavioral state space, that is, desired locomotory patterns, by removing neurons and/or links, and thus potentially rescuing lost function.

The predictions for synthetic essential neuronal pairs and triplets also begin to reveal the functional dependence of particular muscle groups on small sets of neurons. The double ablations show how damage to the network upstream of the muscles (up to Layer 0) can lead to loss of control downstream. The removal of particular pairs reduces the number of independent control signals from the input neurons arriving at Layer 1, which goes on to have an effect on control of the muscles themselves. For the triple ablations, small groups of muscles exist that receive control signals from only a small number of neurons. Ablating fractions of these sets of neurons leads to fewer signals arriving at the muscle cells, and a loss of controllability from external input. Note that it is often not important which three neurons in these sets, just the total of three. This highlights an amount of redundancy, in the control sense, in the network, which provides robustness to failure—it is not until at least three neurons are removed that the effect is felt. The extent of this robustness may also be revealed by the further examination of larger sets of neuronal ablations, which may well lead to larger sets of predictions. Further, each additional ablated neuron corresponds to a maximum reduction in controllability of one muscle cell.

Importantly, the unexpectedly small and precise sets of falsifiable predictions lend themselves well to experimental confirmation. The identification of a small number of muscle cells will allow for insights into the predicted phenotype, to reduce the space of hundreds of locomotor features that tracking software can measure. Cell-specific laser ablation, or advances in genetic ablation techniques allowing one to distinguish between neurons in a class, can be employed. The experiments will still face two major challenges: (a) Making the appropriate strains. Each class of neuron will need a different marker line, so the triple ablation predictions that cover three different classes will need at a minimum three different labeling transgenes with three different colors; (b) Total number of ablations. To properly control the experiments, a mock-ablation would need to be performed for each reporter combination, and to interrogate the predictions for triple ablations, a number of ablation and mock-ablation experiments will need to be performed on combinations of two neurons within the set of three. Finally, we note that a number of the synthetic essential pairs unfortunately contain at least one neuron that lies within a region of the network with only partial (11 pairs) or missing data (19 pairs), and knowledge of network structure is supplemented by inference (Haspel & O’Donovan, 2012). Thus, further improvements to the wiring diagram are warranted prior to experimental testing these particular pairs. The remaining 27 pairs lie in regions with more reliable connectivity maps and represent the most viable experimentally testable predictions. Forty-one of the 46 synthetic essential triplets also lie within well-mapped areas (four triplets contain one neuron with missing connectivity data, and one triplet contains one with partial connectivity data).

Altogether we predicted the existence of 57 synthetic essential pairs of neurons, and 46 synthetic essential triplets, for the behavioral response to gentle touch. Specifically, these are neurons that when ablated individually are not expected to change the worm’s behavior, but when ablated in combination as pairs or triplets are expected to impact features of locomotion elicited by mechanosensory input. Many of the individual neurons are components of larger classes that have long been known to be implicated in locomotion, such as AVB (Chalfie et al., 1985; Piggott, Liu, Feng, Wescott, & Xu, 2011), and a number of motor neurons from the seven major classes (AS, DA, DB, DD, VA, VB, VD; Chalfie et al., 1985; Donnelly, Clark, Leifer, & Pirri, 2013; Haspel & O’Donovan, 2011; Zhen & Samuel, 2015). Yet, these neurons have not been systematically experimentally assessed in smaller groups, and recent evidence suggests that not all components of a class have the same role (Chew et al., 2017). The affected muscles (see Supporting Information, Section III) inform which features are likely to be impacted, but each pair or triplet must be considered individually and further knowledge would be required for a complete characterization of expected phenotype. Indeed, the loss of control over a single muscle may exhibit very subtle phenotypes that differ on a case-by-case basis, and it does not mean the muscle may not be active. The 57 synthetic essential pairs are predicted to affect small sets of muscle cells, in the dorsal and/or ventral mid-posterior region. For the case of the synthetic essential pair {VA09, VA10}, the ventral posterior muscles MVR18 and MVL18 are most likely to incur a loss of control following the ablation (Figure 2C). This allows us to target our queries in precisely this region. Given the location in the body and the connectivity of these motor neurons, we might expect to observe defects in the tail, similar to the DD ablations in Yan et al. (2017). We might also expect defects in ventral motion in this region. Some other synthetic essential pairs, however, contain the command interneuron AVBL. This neuron is part of the “rich club” of densely connected hub nodes (Towlson, Vértes, Ahnert, Schafer, & Bullmore, 2013), and will entail more complex effects because of its connectivity profile. For instance, it is known to play an important role in forward locomotion (Chalfie et al., 1985; Piggott et al., 2011), and this effect may obscure the effects from its role in control with a second neuron. Groups 1 and 2 of the synthetic essential triplets comprise motor neurons that directly innervate muscle cells in the anterior portion of the body, just below the neck. We may speculate that these triple ablations could impact body bending because of their predicted affected muscles, location, and connectivity (Wen et al., 2012). However, caution must be exercised in these behavioral predictions, given the further complexities of and interacting factors within the nervous system. Experiments and/or simulations will ultimately reveal the precise nature of the phenotypes in response to gentle touch induced by the double and triple ablations, with these systematic predictions serving as a guide.

Finally, we also note that the synthetic essential pairs and triplets are all derived from a target control framework in which the input nodes are the mechanosensory neurons ALML, ALMR, and AVM (anterior touch), or PLML and PLMR (posterior touch), and the output nodes are the 95 body wall muscles. We may vary the input and output sets, and this may result in the recovery of different sets of synthetic essential neurons. We verified that the predictions are recapitulated when the inputs are defined by alternative neurons known to elicit a locomotory response (see Supporting Information, Section IV), suggesting that the predictions may be general to locomotion. Another interesting possibility is to vary the set of output nodes. For example, defining these as the motor neurons may elucidate higher order interactions in the control of neuronal motor commands.

## MATERIALS AND METHODS

### The *C. elegans* Wiring Diagram: Data

We base our analyses upon the mapping of the *C. elegans* connectome presented in Varshney, Chen, Paniagua, Hall, and Chklovskii (2011). This wiring diagram comprises 279 nonpharnygeal neurons connected by 2,194 directed synaptic connections and 1,028 reciprocal gap junctions. Nintey-five muscles connect to the nervous system via 552 neuromuscular junctions to 124 motor neurons. See Supporting Information, Section V, for analyses and discussion concerning recent updates to the connectome (Cook et al., 2019).

### Structural Controllability

Following the approach in Yan et al. (2017), we model the nematode nervous system as a directed network whose nodes include neurons and muscles, and whose links represent the electrical and chemical synaptic connections between them, including neuromuscular junctions. Formally, the dynamics of the system composed of *N* neurons and *M* muscles is described by

ż(t)=f(z,v,t), (1)

where ***z***(*t*) = [*z*_1_(*t*), *z*_2_(*t*), …, *z*_*N*+*M*_(*t*)]^*T*^ denotes the states of *N* + *M* nodes at time *t*, ***f***(*) = [*f*_1_(*), *f*_2_(*), …, *f*_*N*+*M*_(*)]^*T*^ captures the nonlinear dynamics of each node, and ***v***(*t*) = [*v*_1_(*t*), *v*_2_(*t*), …, *v*_*S*_(*t*)]^*T*^ represents the external stimuli applied to the S touch receptor neurons. Assuming that in the absence of additional stimuli the nervous system is at a fixed point z*, where ***f***(z*, v*, *t*) = 0, and using ***x***(*t*) = ***z***(*t*) − z* and ***u***(*t*) = ***v***(*t*) − v*, Equation 1 can be linearized, obtaining

$$\left\{\begin{array}{c} \dot{x}\left(t\right)=Ax\left(t\right)+Bu\left(t\right),\\ y\left(t\right)=Cx\left(t\right), \end{array}\right.$$ (2)

where *A* ≡ $\frac{\partial f}{\partial z}{|}_{z^{*},v^{*}}$ corresponds to the adjacency matrix of the connectome, with nonzero elements *A*_*ii*_ that represent the nodal dynamics of node i; the input matrix *B* ≡ $\frac{\partial f}{\partial v}{\left|\right|}_{z^{*},v^{*}}$ represents the receptor neurons on which the external signals are imposed, for example, ALML/R and AVM for anterior gentle touch; and the vector *y*(*t*), selected by the output matrix *C*, represents the states of the M muscle cells. In other words, the response of *C. elegans* to external stimuli can be formalized as a target control problem (Gao et al., 2014), asking whether the inputs received by receptors in *B* can control the state of the muscles listed in *C*. The muscles are controllable if, with a suitable choice of inputs ***u***(*t*), they can move in any desired manner, that is, ***y***(*t*) can reach an arbitrary position of the M-dimensional state space (Coron, 2009). To determine this, we consider the controllability matrix, given by ***K*** = [*CB*, *CAB*, *CA*^2^*B*, …, *CA*^*N*+*M*−1^*B*]. Kalman’s criterion (Kalman, 1963) tells us that the System 2 is deemed structurally controllable if *rank* ***K*** = *M*; this translates to the situation that all *M* muscles are controllable via signals from the input sensory neurons. Moreover, *rank* ***K*** is equal to the number of controllable muscles, thus providing a way to measure the level of controllability (Yan et al., 2017).

## ACKNOWLEDGMENTS

We thank Yee Lian Chew, István Kovács, Denise Walker, and William Schafer for thoughtful and valuable discussions.

## SUPPORTING INFORMATION

Supporting information for this article is available at https://doi.org/10.1162/netn_a_00115. The *C. elegans* wiring diagram is publicly available online (Cook et al., 2019; Varshney et al., 2011), and details of all results are either provided as Extended Data or can be found at https://github.com/EmmaTowlson/c-elegans-control (Towlson, 2019). All analyses were completed in Python using scripts available at Towlson (2019).

## AUTHOR CONTRIBUTIONS

Emma Towlson: Conceptualization; Formal analysis; Funding acquisition; Investigation; Methodology; Visualization; Writing - Original Draft; Writing - Review & Editing. Albert-László Barabási: Conceptualization; Funding acquisition; Supervision; Writing - Review & Editing.

## FUNDING INFORMATION

Albert-László Barabási, National Science Foundation (http://dx.doi.org/10.13039/100000001), Award ID: 1734821. Albert-Láslzó Barabási, National Science Foundation, Award ID: 1735505 - INQUIRE. Albert-László Barabási, Horizon 2020 Framework Programme (http://dx.doi.org/10.13039/100010661). Albert-László Barabási, European Research Council (http://dx.doi.org/10.13039/501100000781), Award ID: 810115 - DYNASNET.

## TECHNICAL TERMS

Synthetic lethality: Biological phenomenon whereby the deletion of individual genes is tolerated, but their simultaneous deletion leads to cell or organism death.
Target control: Network control framework that requires the control only of a set of output nodes central to a required task.
Gentle touch response: The reversal locomotion performed by *C. elegans* upon gentle stimuli to the mechanosensory neurons AVM, ALM, and/or PLML.
Structural control: Network control approach that assesses the role of the wiring diagram (presence/absence of a link) in network controllability.
Synthetic essential: Two or more neurons that are each individually nonessential for a given behavior, but their combination is essential.
Simplicial complex: A system comprising nodes connected by simplexes.
Simplex: The simultaneous interaction between three or more nodes: an extension of the dyadic edge to polyadic relationships.
