# Supplementary material for: Synthetic ablations in the C. elegans nervous system
Source: Netw Neurosci. 2020 Mar 1;4(1):200–16. doi: 10.1162/netn_a_00115 (PMC7055645; doi:10.1162/netn_a_00115)
Supplement: Supplementary file 1 [file netn-04-200-s001.pdf]

Towlson, E., & Barabási, A. (2020). Supporting information for "Synthetic ablations in the C. elegans nervous system." *Network Neuroscience*, *4*(1), 200-216. https://doi.org/10.1162/netn_a_00115

Synthetic ablations in the *C. elegans* nervous system

Supplementary Information

Emma K. Towlson and Albert-László Barabási

I. Detailed results: Double ablations

As discussed in the main text, we recover 57 synthetic essential pairs, with only three Mechanisms of loss of control over the body-wall muscle cells following external input to the mechanosensory neurons implicated in gentle touch. We also identify a small number of double ablations which, while expected to result in such a loss of control due to the involvement of one or more essential single neurons, do not result in the precise level of controllability loss expected from the essential single neuron(s) alone; see Tables S1 and S4, and Extended Data 1. Most notably, we find only one example of enhancement of control loss. A pair comprising one singly essential neuron and one non-essential neuron ($E_{single}N_{single}$) would be expected to result in the loss of fine control over one muscle cell, due to the essential neuron. We find one such pair where the value of loss of control increases to two muscle cells: {AS11, RID}. Here AS11 is essential alone, and RID is non-essential.

We also note four instances where the loss of control is diminished from the expectation. We would expect a pair comprising two singly essential neurons ($E_{single}E_{single}$) to result in the loss of fine control over two muscle cells if we were to simply sum the effects – one muscle for each ablated neuron. We recover four such pairs which only lead to a loss of control over *one* muscle cell: {AVAL, AS08}, {AVAR, AS08}, {AVAL, DA07}, and {AVAR, DA07}. This can be

explained by an overlap in network neighbourhood; the same muscle cells are affected by the ablation of each individual neuron, so the impact of their simultaneous removal is contained to the same cells.

II. Detailed results: Triple ablations

We identify four groups of synthetic essential triplets. The network effects behind Groups 1 and 2 are shown in Figure 4(b) and (c). When the three neurons in Group 3 {SMBDL, SMDDL, SMDDR} are ablated, the muscle MDR06 is disconnected entirely from the network – see Figure S1. We do not show Group 4 as its constituents are not localised in the network. In addition to these groups, we find a number of predictions for enhancement of control loss. These triplets contain singly essential or synthetic essential pairs, but are predicted to lead to a greater reduction in controllability than expected from the summation of effects alone; see Tables S2 and S5. Specifically:

(i) A triplet comprising a synthetic essential pair and an essential single neuron ($E_{pair}E_{single}$) would be expected to result in the loss of fine control over two muscle cells in the response to gentle touch in the case of a simple summation of effects – one for the pair, and one for single neuron. We identify one such triplet, {PDA, AS11, RID}, which results in the loss of control over *three* muscle cells. This is consistent with the observation that {PDA,RID} is a synthetic essential pair, and as noted above the ablation of {AS11,RID} leads to a loss of control over two muscle cells.

(ii) A triplet comprising two singly essential neurons and one non-essential neuron ($E_{single}E_{single}N_{single}$) would also be expected to result in the loss of fine control over two muscle cells in the response to gentle touch, one for each essential neuron. We identify 18 triplets where this loss of control is increased to three muscle cells. All of these triplets contain the pair {AS11, RID} and one further neuron (see Extended Data 2), again consistent with the enhancement of loss of control found in the double ablations.

(iii) A triplet comprising one single essential neuron and two non-essential neurons (EsingleNsingleNsingle) would be expected to lead to a loss of control in one muscle cell in the response to gentle touch. We find 1363 triplets where this loss is predicted to be increased to two muscle cells (see Extended Data 2).

Finally, we predict a number of triple ablations will result in a smaller reduction in controllability in the response to gentle touch than expected by the summation of the effects of parts. This is due to an overlap in the neurons and muscles involved in each case. In summary, these cases are: 149 triplets of the form $3E_{pair}$; 16 of the form $2E_{pair}$; 43 of the form $E_{pair}E_{single}$; 68 of the form $E_{single}E_{single}E_{single}$; and 1036 of the form $E_{single}E_{single}N_{single}$. See also Table S2.

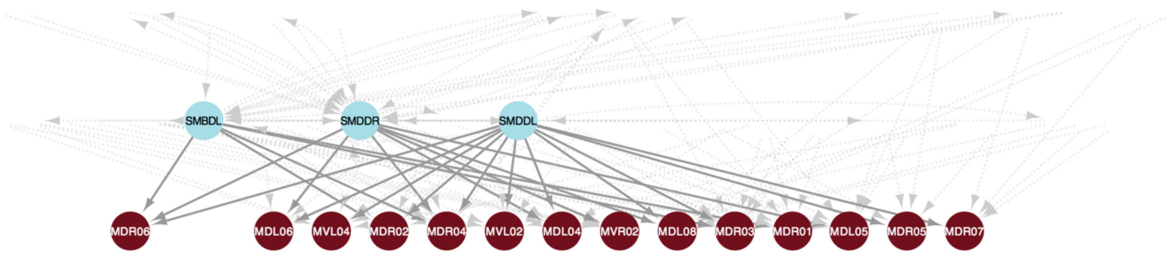

Figure S1: Group 3. The removal of {SMBDL, SMDDL, SMDDR} disconnects the muscle cell MDR06.

III. Affected muscles

The structural controllability framework provides a deterministic number of controllable muscles, but there are in general multiple configurations of muscles that could comprise these controllable cells (Towlson et al., 2018). Therefore, while we find 89 independently controllable muscles in the healthy worm, the precise set of 89 muscles is not unique: there are multiple solutions to the control problem (Jia & Barabási, 2013), each of which give rise to the same level of controllability. By cataloguing these independent solutions, we can assign a probability to which muscles are more likely to experience a reduction in control in the response to gentle touch. Specifically, for each ablation of pairs or triplets of neurons, we numerically obtained the probability pattern of each muscle losing its controllability in the context of receiving mechanosensory stimuli, and compared this pattern to that of the healthy worm. We obtained these patterns through 1000 iterations of the structural controllability analysis. The difference between the two probability patterns reveals which muscles are affected most strongly by the ablation. The muscle patterns tend to be highly spatially co-localised, offering quite specific predictions pertaining to expected phenotypes in future experiments.

Indeed, the synthetic essential pairs are predicted to affect only a small set of muscles in the ventral posterior section of the body. Figure S2 shows three exemplary pairs, and the consistent region predicted to experience a loss of control. Probability patterns for all predictions can be found at https://github.com/EmmaTowlson/c-elegans-control.

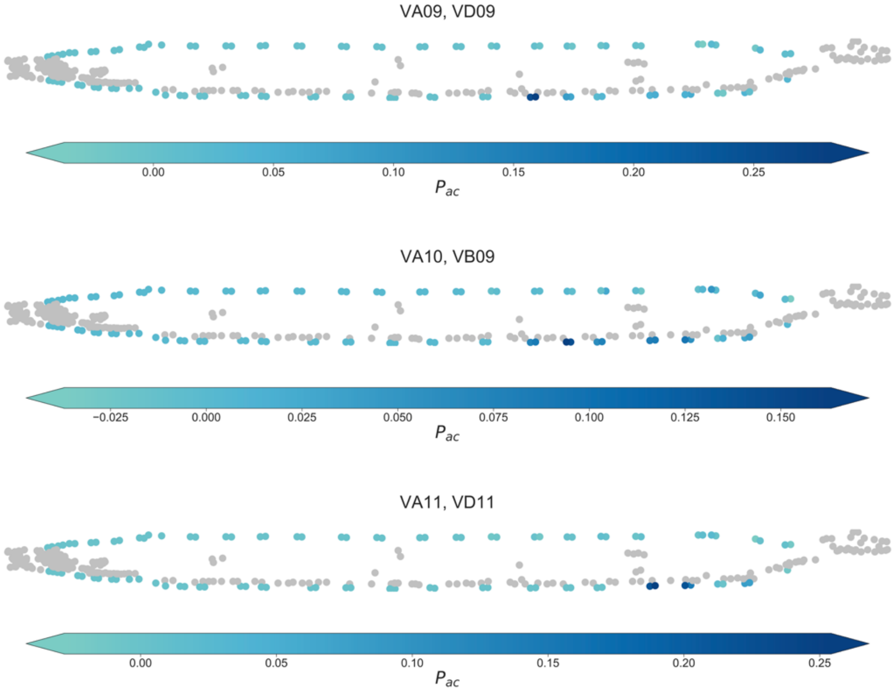

***Figure S2**: Affected muscles for synthetic essential pairs*. The probability of ablation-induced loss of control in the response to gentle touch over each muscle is shown in the body of *C. elegans* following ablation of the pair {VA09, VD09} (top), {VA10, VB09} (middle), and {VA11, VD11} (bottom). Muscles most likely to lose control are coloured dark blue, and the least likely to lose control in green. The locations of neuron cell bodies are shown in grey. A similar area in the ventral posterior section of the body is affected most strongly in each case.

IV: Alternative input neurons

The analysis conducted in the main paper considers the locomotory response to gentle touch on the anterior and posterior body, which translates to the input neurons in the vector B in Equation 2, being {ALML, ALMR, AVM} and {PLML, PLMR} respectively. We considered three further sets of sensory neurons for the input set, each known to elicit a locomotory response upon stimulation: {FLPL, FLPR}, {PVDL, PVDR}, and {ASHL, ASHR}. In each case, we ablated *in silico* the 58 synthetic essential pairs and 1327 synthetic triplets that we uncovered in our original analysis as resulting in a greater loss of controllability than expected by the summation and/or overlap of lower order results. We recovered precisely the same findings in terms of quantifying the loss of controllability, suggesting, as per our original study (Yan et al., 2017), that these control properties are more general to locomotion in *C. elegans* rather than specific to just one behaviour. In other words, the uncovered synthetic pairs and triplets are not just important for the response to gentle touch, but also for other locomotion-based behaviours.

V. The *C. elegans* wiring diagram: alternative data

The analyses in the main text are based upon the mapping of the *C. elegans* connectome presented in (Varshney, Chen, Paniagua, Hall, & Chklovskii, 2011). The imperfections in the wiring diagram have recently come under scrutiny from the community (Haspel & O'Donovan, 2012; Towlson et al., 2018; Xu et al., 2013), and indeed the original EM images were recently reexamined, resulting in a new wiring diagram with significant differences in connectivity (Cook

et al., 2019). As such, we reexamined our main findings on this version. The new connectome has some significant differences which must be addressed first:

(i) Electrical coupling between the body wall muscle cells is modelled in the form of bidirectional edges between neighbouring muscle cells. This coupling is not modelled in the wiring diagram used in the main text. It is important to note that the structural control framework relies on a path-based approach, and incorporating electrical coupling in this manner simply leads to the conclusion that all muscle cells are always controllable: when a control signal reaches any muscle cell, it can continue along the paths between the muscles themselves indefinitely. There is no current methodology to appropriately account for the difference in the properties of different connection types (Towlson et al., 2018), so we removed these coupling links.

(ii) This dataset is enriched with the connectivity of numerous end organs not present in the earlier wiring diagram. To facilitate a direct comparison with our main results, and to remain focused on control of locomotion as driven by the body wall muscles, we removed the extra end organs. This ensured the same set of 95 muscle cells as the target nodes in the structural control problem.

(iii) Finally, there are a significant number of extra links in the updated network. At this high density, we recover fewer results from the control framework (see Table S6). Any inevitable mapping errors are more likely to lie within the weakest connections, and they are more likely to be sites of variation between individuals. Therefore it is reasonable to hypothesise that a robust control structure should exist among the stronger, or more reliable, links. We systematically pruned the weakest links, i.e.

those with the fewest synapses between neuron pairs, from the network. We considered thresholds of $\square = 0 - 3$ neurons as criteria for the presence or absence of an unweighted link. For $\square = 0$ all links remain, and for $\square = 3$ links with fewer than 3 synapses were removed.

These processing steps provided us with a connectome with $N + M = 375$ nodes (the 374 neurons and muscles in the original connectome, plus VC06, a neuron which is disconnected in the previous dataset) and $L = 6022$ directed unweighted links. With an increasing threshold this reduced the number of links to 4124 ($\tau = 1$), 3068 ($\tau = 2$), and 2318 ($\tau = 3$).

Firstly, we examined whether or not our original predictions for single neuron ablations were robust to the differences in the two wiring diagrams; our findings can be found in Table S6. We recover the most complete set of predictions at a threshold of three synapses, i.e. where links with only one or two synapses are pruned from the network. At this threshold, we find 91 controllable muscles in the healthy worm, and recover all predictions from the original analysis (Yan et al., 2017) plus an extra twelve neurons. These twelve neurons comprise eleven ventral motor neurons plus PDA, and offer potential further single-cell predictions for experimental testing.

We focused our next analyses on the connectome with the greatest correspondence with our original predictions – that with the threshold of 3 synapses. We repeated the structural control analysis for the 57 synthetic essential double ablations and the 46 synthetic essential triple ablations. We observe large agreement with the synthetic essential pairs: 53 also lead to a

reduction in body-wall muscle controllability from external mechanosensory input in this connectome, while the final 4 do not. We find less consistency when examining the synthetic essential triplets: only 17 of the 46 are predicted to reduce control. While the two connectomes initially recover similar results for the control analysis, as we move to higher order interactions we encounter more differences. Given the large differences between the two wiring diagrams this is not surprising, as the finer details of the network structure become more important with the removal of more neurons. More accurate and modern maps will improve the accuracy and completeness of the predictions higher order interactions. Nevertheless, these results are encouraging. The consistency in the double ablation predictions in particular highlights a large degree of robustness of the control organisation to rewired links.

References

Cook, S. J., Jarrell, T. A., Brittin, C. A., Wang, Y., Bloniarz, A. E., Yakovlev, M. A., … Emmons, S. W. (2019). Whole-animal connectomes of both Caenorhabditis elegans sexes. *Nature*, *571*, 63–71.

Haspel, G., & O'Donovan, M. J. (2012). A connectivity model for the locomotor network of *Caenorhabditis elegans*. *Worm*, *1*(2), 125–128. https://doi.org/10.4161/worm.19392

Jia, T., & Barabási, A. L. (2013). Control capacity and a random sampling method in exploring controllability of complex networks. *Scientific Reports*, *3*, 2354. https://doi.org/10.1038/srep02354

Towlson, E. K., Vértes, P. E., Yan, G., Chew, Y. L., Walker, D. S., Schafer, W. R., & Barabási, A.-L. (2018). *Caenorhabditis elegans* and the network control framework—FAQs. *Philosophical Transactions of the Royal Society B: Biological Sciences*, *373*(1758). Retrieved from http://rstb.royalsocietypublishing.org/content/373/1758/20170372.abstract

Varshney, L., Chen, B., Paniagua, E., Hall, D., & Chklovskii, D. (2011). Structural properties of the *Caenorhabditis elegans* neuronal network. *PLoS Comput. Biol.*, *7*(2), e1001066.

Xu, M., Jarrell, T. A., Wang, Y., Cook, S. J., Hall, D. H., & Emmons, S. W. (2013). Computer Assisted Assembly of Connectomes from Electron Micrographs: Application to Caenorhabditis elegans. *PLoS ONE*, *8*(1). https://doi.org/10.1371/journal.pone.0054050

Yan, G., Vértes, P. E., Towlson, E. K., Chew, Y. L., Walker, D. S., Schafer, W. R., & Barabási, A.-L. (2017). Network control principles predict neuron function in the *Caenorhabditis elegans* connectome. *Nature*, *550*(7677), 519–523. https://doi.org/10.1038/nature24056

Supplementary tables

	Reduction (-2)	Reduction (-1)	No effect
$E_{single}E_{single}$	186	4	0
$E_{single}N_{single}$	**1**	5179	0
$N_{single}N_{single}$	0	**57**	33353

Table S1: **Double ablation predictions and amount of reduction in control.** As per Table 1, a selected pair of neurons may comprise two individually essential neurons (E_{single}), one essential and one non-essential neuron (N_{single}), or two non-essential neurons. A loss of control over one (-1) or two (-2) muscles is predicted from double ablations. Synthetic essential pairs with no overlap with single ablation predictions are coloured **red**. One pair is predicted to lead to a greater loss of control than explained by the presence of a single essential neuron alone, coloured in **blue**. Predictions commensurate with the simple summation and/or overlap of effects of single ablations are coloured black. Totals exclude the trivial case of removal of all input neurons.

		Reduction (-3)	Reduction (-2)	Reduction (-1)	No effect
Pairs	$3E_{pair}$	0	149	0	0
	$2E_{pair}$	0	0	16	0
	$E_{pair}E_{single}$	**1**	0	43	0
	$E_{pair}N_{single}$	0	0	14170	0
No pairs	$E_{single}E_{single}E_{single}$	1072	68	0	0
	$E_{single}E_{single}N_{single}$	**18**	48156	1036	0
	$E_{single}N_{single}N_{single}$ – no P	0	**1363**	666813	0
	$N_{single}N_{single}N_{single}$– no P	0	0	**46**	2847827

Table S2: **Triple ablation predictions and amount of reduction in control.** As per Table 2, a selected triplet of neurons is comprised of combinations of individually essential neurons (E_{single}), individually non-essential neurons (N_{single}), and synthetic essential pairs of neurons (E_{pair}). A loss of control over one (-1), two (-2), or three (-3) muscles is predicted from triple ablations. Synthetic essential triplets with no overlap with single ablation predictions, and not containing a

complete pair of synthetic essential neurons, are coloured **red**. Triplets predicted to lead to a greater loss of control than explained by the presence of one or more essential neurons or neuron pairs alone are coloured in **blue**. Predictions commensurate with the simple summation and/or overlap of effects of single and/or double ablations are coloured black. Totals exclude the trivial case of removal of all input neurons.

AS08
AS09
AS10
AS11
AVAL
AVAR
DA07
DA08
DA09
DB05
DB06
DB07
DD04
DD05
DD06
PDB
VA12
VB11
VD12
VD13

Table S3: **Neuronal components of the single ablation predictions.** Those in *italics* have been experimentally verified (1). All ablations are predicted to lead to the loss of independent control over one muscle cell.

Synthetic essential pair		Mechanism
AS07	DB04	Mechanism 1
AS07	DA06	
DA06	DB04	
PDA	RID	
VA09	VB09	
VA09	VD09	
VA09	VB08	
VA09	VB10	
VA09	VD11	
VA09	VD10	
VA09	VA10	
VA09	VA11	
VA10	VB09	
VA10	VD09	
VA10	VB08	
VA10	VB10	
VA10	VD11	
VA10	VD10	
VA10	VA11	
VA11	VB09	
VA11	VD09	
VA11	VB08	
VA11	VB10	
VA11	VD11	
VA11	VD10	
VB08	VB09	
VB08	VD09	
VB08	VB10	
VB08	VD11	
VB08	VD10	
VB09	VD09	
VB09	VB10	
VB09	VD11	
VB09	VD10	
VB10	VD09	
VB10	VD11	
VB10	VD10	
VD09	VD11	

VD09	VD10	
VD10	VD11	
AVBL	DVB	Mechanism 2
AVBL	VB09	Mechanism 3
AVBL	VA09	
AVBL	VD09	
AVBL	VB08	
AVBL	VB10	
AVBL	VD11	
AVBL	VD10	
AVBL	VA11	
DVB	VB09	
DVB	VA09	
DVB	VD09	
DVB	VB08	
DVB	VB10	
DVB	VD11	
DVB	VD10	
DVB	VA11	

Table S4: **Neuronal components of the double ablation predictions**. Synthetic essential pairs arise from three mechanisms (see Figure 2(a) and Figure 3).

Synthetic essential triplet			Group
AS03	DA03	DD02	Group 1
AS03	DA02	DD01	
AS03	DA03	DD01	
AS03	DA03	DA02	
AS03	DA02	DD02	
AS03	DD01	DD02	
DA02	DD01	DD02	
DA03	DA02	DD02	
DA03	DA02	DD01	
DA03	DD01	DD02	
DB02	AS03	DD02	
DB02	DA03	DD01	
DB02	AS03	DD01	

DB02	DA02	DD02	
DB02	DA03	DD02	
DB02	AS03	DA03	
DB02	DA02	DD01	
DB02	DD01	DD02	
DB02	DA03	DA02	
DB02	AS03	DA02	
VA03	VD03	PVNL	Group 2
VB02	VD03	PVNL	
VB02	VA03	PVNL	
VA03	VD03	VB02	
SMDDL	SMDDR	SMBDL	Group 3
DA06	AVBR	AVBL	Group 4
DA06	AVBR	VA11	
DA06	DVB	AVBR	
VB08	DA06	AVBR	
VB10	DA06	AVBR	
VD09	DA06	AVBR	
VD10	DA06	AVBR	
VD11	DA06	AVBR	
VA09	DA06	AVBR	
VB09	DA06	AVBR	
DB04	AVBR	AVBL	
DB04	AVBR	VA11	
DB04	DVB	AVBR	
DB04	VB08	AVBR	
DB04	VB10	AVBR	
DB04	VD09	AVBR	
DB04	VD10	AVBR	
DB04	VD11	AVBR	
VA09	DB04	AVBR	
VB09	DB04	AVBR	
PDEL	VB10	AVFR	

Table S5: **Neuronal components of the triple ablation predictions.** Synthetic essential triplets occur in four distinct groups (see Figure 4(a)).

**Threshold τ (# synapses)**			
**1**	**2**	**3**	**4**
AS08	*AS08*	*AS08*	*AVAL*
AS09	*AS09*	*AS09*	*AVAR*

AS10	*AS10*	*AS10*	*DA07*
AS11	*AS11*	*AS11*	*DA08*
DA07	*DA07*	*AVAL*	*DA09*
DA08	*DA08*	*AVAR*	*DB05*
DA09	*DA09*	*DA07*	*DD04*
DB05	*DB05*	*DA08*	*DD05*
DB06	*DB06*	*DA09*	*DD06*
DB07	*DB07*	*DB05*	PDA
DD04	*DD04*	*DB06*	PVPL
DD05	*DD05*	*DB07*	VA11
DD06	*DD06*	*DD04*	*VA12*
PDA	PDA	*DD05*	VB07
PDB	*PDB*	*DD06*	VB08
		PDA	VB09
		PDB	VB10
		VA09	*VB11*
		VA10	VD08
		VA11	VD09
		VA12	VD10
		VB07	VD11
		VB08	*VD12*
		VB09	*VD13*
		VB10	
		VB11	
		VD08	
		VD09	
		VD10	
		VD11	
		VD12	
		VD13	

Table S6: **Neuronal components of the single ablation predictions for a recently reanalysed wiring diagram.** Those in *italics* match the findings in the original connectome as per Table S3. All ablations are predicted to lead to the loss of independent control over one muscle cell.
